# Multifaceted human gut microbiome data associated with health and nutrition

**DOI:** 10.3389/fmicb.2026.1722500

**Published:** 2026-02-16

**Authors:** Lucia Maisto, Claudia Telegrafo, Francesco Rubino, Monica Santamaria, Maria H. Traka, Apollonia Tullo, Jildau Bouwman, Elisabetta Sbisà, Bachir Balech

**Affiliations:** 1 Institute of Biomembranes, Bioenergetics and Molecular Biotechnologies, Consiglio Nazionale delle Ricerche (IBIOM-CNR), Bari, Italy; 2 Institute for Biomedical Technologies, Consiglio Nazionale delle Ricerche (ITB-CNR), Bari, Italy; 3 Department of Soil, Plant and Food Sciences (Di.S.S.P.A.), University of Bari, Bari, Italy; 4 Food and Nutrition National Bioscience Research Infrastructure, Quadram Institute Bioscience, Norwich Research Park, Norwich, United Kingdom; 5 Netherlands Organisation for Applied Scientific Research (TNO), Microbiology and Systems Biology, Leiden, Netherlands

**Keywords:** database, diet, disease, dysbiosis, FAIR, metagenomics

## Abstract

The microbiome, also considered the hidden organ, is a fundamental ecosystem directly associated with the disease and health status of the human body. With the availability of high-throughput DNA sequencing technologies, a growing number of studies from clinical and experimental (observation and intervention) samples are constantly revealing new findings on the relationship between human organs and their microbiomes. In such a context, diet and nutrition are among the key factors influencing microbiome composition, richness, and functional behavior. In this review, we illustrate how microbiome-related data and associated metadata are in recent times scattered across primary and specialized databases with different levels of curation, annotation, and standardization, limiting, to some extent, the possibility of deep data discovery, reuse, alignment, and harmonization. Therefore, we describe the way Findable, Accessible, Interoperable, and Reusable (FAIR) data principles would enhance the onset of novel scientific hypotheses and potential microbiome-targeted therapies by improving the standardization policies in data sources. Accordingly, using advanced semantic classification and data mining technologies based on suitable and comprehensive ontologies, annotations of studies present in source databases or in scientific literature would further improve the data and metadata enrichment, integration and alignment relevant to microbiome data associated with health, disease and nutrition.

## 1 Introduction

The microbiota is a complex and dynamic ecosystem essential for health as it interacts with nearly every aspect of human physiology (Khalil et al., 2024; Origüela and Lopez-Zaplana, 2025). It is often referred to as the “hidden organ” within an organ, highlighting its critical roles in health and disease (Hou et al., 2022). Growing evidence links the human microbiota to mental and physical wellbeing through the gut–brain axis, a bidirectional network involving neural, immune, and endocrine pathways regulating both immunity and inflammation (Martin et al., 2018; Morais et al., 2021; Origüela and Lopez-Zaplana, 2025). Similarly, the skin, lung, vaginal, and oral microbiota, which vary by niche, regularly influence directly and indirectly the response to several human body disorders (Yamashita and Takeshita, 2017; Mithradas et al., 2024; Kreouzi et al., 2025; Zhou et al., 2025).

Although a great number of scientific studies have been carried out, multiple aspects of human health in relation to food and its correlation with microbiome composition and function remain to be discovered. Such complexity can only be addressed through the availability and reusability of a suitable amount of high-quality microbiome-derived data, including nucleic acids (DNA/RNA sequences), capabale of enhancing, maximizing, and standardizing scientific research and data elaboration and modeling. Accordingly, to achieve such a purpose, public DNA/RNA sequence databases adopting appropriate policies for sustainable data management, including structure, sharing protocols, accessibility, and reusability [i.e., (Zhao et al., 2023), Findable, Accessible, Interoperable, Reusable (FAIR) Wilkinson et al., 2016],^1^ have become a fundamental aspect.

In this study, we explore the main features describing data type and availability in public databases related to the human gut microbiome in association with general health and food intake. The main microbiome data sources were explored, including their adherence to standards, open science, and FAIR data principles. Recommendations on the proper use of such data and the possibility of aligning studies and datasets across resources are also provided.

## 2 Eubiosis and dysbiosis

The gut microbiome significantly influences the health and disease status of each individual as it contributes to several interactions with near or distant organs. Scientific evidence links the effect of microbiome state to numerous important human diseases, such as cardiovascular diseases, type 2 diabetes, neurological development disorders, autoimmune diseases, and colon and colorectal cancer. The absence or the severity of such diseases/disorders is determined by two main classes of microbiome features called eubiosis or dysbiosis (Iebba et al., 2016; Yu et al., 2022; Goudman et al., 2024; Hamjane et al., 2024; Díez-Madueño et al., 2025).

Eubiosis is essential to maintain a healthy gut. Despite its importance, eubiosis remains partially understood, with ongoing research exploring the microbiome–host relationship and its therapeutic potential (Hou et al., 2022). It is a balanced gut microbiota state where diverse microbial communities interact harmoniously to support the host’s health (Chen et al., 2024). This equilibrium is achieved through the interplay of key bacterial taxa, including *Firmicutes*, *Bacteroides*, *Actinobacteria,* and *Proteobacteria*, which collectively regulate essential processes such as nutrient metabolism and immune modulation (Afzaal et al., 2022). For instance, the small intestine hosts *Enterobacteriaceae* for nutrient absorption, while the colon, dominated by *Bacteroidetes* and *Firmicutes*, is the center of microbial activity (Rinninella et al., 2019). The gut microbiota shapes immune responses by helping immune cells distinguish commensals from pathogens, for instance, by activating NF-κB signaling via Toll-like receptors and promoting tolerance through Treg cell stimulation (Al-Rashidi, 2022; Brockmann et al., 2023). Moreover, a healthy microbiota prevents pathogen colonization by depleting essential nutrients and forming physical biofilm barriers, while simultaneously using specialized enzymes to metabolize complex carbohydrates reserved for their prebiotic potential (Ma et al., 2015; Bedu-Ferrari et al., 2024). Additionally, commensals secrete antimicrobials such as bacteriocins and metabolites (e.g., reuterin produced by *Lactobacillus reuteri*) that inhibit pathogens and potentially prevent colon tumorigenesis (Bell et al., 2022). Recent advances have highlighted the impact of gut microbiota and oral probiotics supplementation on defense responses also against viral infections, including respiratory viruses, through the modulation of the pulmonary immune response of the gut–lung axis (Andrade et al., 2022; Chen N. et al., 2025; Liu et al., 2025).

On the other hand, dysbiosis, referring to an imbalanced gut microbiota, is characterized by reduced microbial diversity, promoting pathogenic overgrowth and leading to metabolic and immune dysfunctions (Chen et al., 2024). Emerging evidence links dysbiosis to colorectal cancer, as increased permeability allows pro-inflammatory metabolites to promote inflammation, DNA damage, and tumorigenesis (Artemev et al., 2022). For instance, the overgrowth of specific bacteria such as *Escherichia coli* and *Bacteroides fragilis* contributes to genotoxin production (Li et al., 2021; Zhao et al., 2023). A dysbiotic microbiota, marked by a higher Firmicutes-to-Bacteroidetes ratio, alters lipid and glucose metabolism and compromises intestinal barrier integrity and function, leading to systemic inflammation and insulin resistance, both of which are associated with obesity and type 2 diabetes (Brunkwall and Orho-Melander, 2017; Gomes et al., 2018), and a reduction in butyrate production, contributing to glucose intolerance (Gomes et al., 2018). Chronic intestinal disorders are also associated with reduced short-chain fatty acid (SCFA) production and disrupted tight junctions, which compromise epithelial integrity and trigger excessive immune activation that drives inflammatory disease progression (Qiu et al., 2022), while also negatively influencing the correct gut–brain axis communication (Mehta et al., 2025; Yassin et al., 2025). Restoring SCFA-producing bacterial genera, species, or strains may support epithelial repair and consequently dampen inflammatory responses (Effendi et al., 2022) and fix brain functions (Mehta et al., 2025; Yassin et al., 2025).

The proof of association between pathological conditions and microbiome composition and function is expected to grow continuously as related data are becoming increasingly available in an open-science context. As stated above, potential discoveries on specific taxonomic or functional profiles—including, eubiotic or dysbiotic taxa fingerprints connected to certain health status—can be recovered from relevant scientific studies. However, similar assumptions should be drawn only from comprehensive and combined microbiome datasets supported with accurate metadata annotation and curation, based on controlled vocabularies or specific ontologies, permitting an advanced level of harmonization across data sources.

## 3 Microbiome-derived DNA/RNA sequence data sources

Advances in sequencing technologies [e.g., next-generation sequencing (NGS)] have promoted deeper exploration of microbial communities and their relative functional profiles in environmental samples (i.e., human organs) through the application of metagenomics, metatranscriptomics, and metabarcoding (Franzosa et al., 2014; Arıkan and Muth, 2023). This advancement has resulted in a vast amount of sequence data being stored in public primary, specialized, or specific project-dedicated databases with different levels of analysis layers, data curation, metadata enrichment, and data sustainability plans.

Primary public databases such as the *Sequence Read Archive (SRA)* (Katz et al., 2022), managed by the *National Center for Biotechnology Information* (NCBI), and the *European Nucleotide Archive* (ENA) (O’Cathail et al., 2025), managed by EBI, primarily serve as storage and organization facilities for raw sequencing data, including microbiome-related NGS data (i.e., metagenomics and metabarcoding). In parallel, specialized databases have been developed to offer additional services such as advanced analysis, data curation, and annotation. These databases make use of the data provided by the users or those available in primary databases or in related scientific papers and provide state-of-the-art bioinformatics tools and user-friendly interfaces for microbial communities profiling. In this context, adherence to FAIR data principles, ensuring accessibility, interoperability, and re-usability, provides unprecedented benefits. However, FAIRness fulfillment does not guarantee enough information background for scientific use, as the inclusion of enriched metadata is not always mandatory.

In terms of data volume, annotation, wide content, and FAIR compliance, it is worthy to mention three main specialized data sources, namely (i) MGnify, developed by EBI-Metagenomic (Richardson et al., 2023), (ii) MG-RAST (Meyer et al., 2008), developed by the University of Chicago (Keegan et al., 2016), and (iii) JGI IMG/M – /VR, developed by the Joint Genome Institute of California (Chen et al., 2023; Mukherjee et al., 2025).

Although many other microbiomic data sources are of pronounced importance, their use might be more limited due to their specific, clear-cut content objectives, their reduced annotation level, or their outdated metadata, as they are related to closed scientific projects. Nevertheless, one of the most influential but archived initiatives is the *Human Microbiome Project* with its HMP data portal (Turnbaugh et al., 2007), which generated, over several years, a large volume of data and important scientific papers regarding the characterization of the human microbiome and its role in health and disease (Integrative HMP (iHMP) Research Network Consortium, 2019). Several databases make use of the data produced by HMP and integrate it into their own datasets.

As illustrated in Table 1, some resources target microbiomes from multiple organs or diseases [GIMICA (Tang et al., 2021), DISBIOME (Janssens et al., 2018), gcMeta (Shi et al., 2019), Microbiome database (MDB),^2^ Qiita (Gonzalez et al., 2018), curatedMetagenomicData (Pasolli et al., 2017), and Phenotype Database (van Ommen et al., 2010)], while others focus on single organ mainly the gut such as gutMDisorder (Qi et al., 2022), GMrepo (Dai et al., 2022), Human Gut Microbiome Atlas,^3^ NIBN JMD (Chen Y.-A. et al., 2025). A majority of these databases offer both raw data (or links to raw data) and associated metadata, taxonomic and functional microbiome profiles, and an integrated framework of scientific research results elaborated according to a specific bioinformatics pipeline and experimental parameters. Moreover, some resources, such as Qiita and curatedMetagenomicData, provide ready-to-use reference backbone data in standard formats (R or Python objects), which can be incorporated into an in-house data analysis routine.

**Table 1:** Main features of human microbiome molecular data sources. Database type, content, data, and metadata formats, and the last update are shown.

Database name	Type	Data retrieval protocol	Link	Data content	Data and metadata format/standard	Last update	Notes	References
NCBI SRA	P	Api/Web/ftp	https://www.ncbi.nlm.nih.gov/sra	Global Multiple organs	Fastq, BAM, JSON	2025	Important to obtain raw data and metadata. Easy to include in data analysis pipelines.	Katz et al. (2022)
ENA	P	Api/Web/ftp	https://www.ebi.ac.uk/ena	Global Multiple organs	Fastq, BAM, CRAM, JSON	2025	Important to obtain raw data and metadata. Easy to include in data analysis pipelines.	O’Cathail et al. (2025)
Mgnify	S	Api/Web	https://www.ebi.ac.uk/about/teams/microbiome-informatics/mgnify/	Multiple organs Health—disease Diet	Fasta, JSON, csv	2025	Wide range of studies. Easy to use in standardized bioinformatics pipelines. Metadata is sometimes missing or not complete.	Richardson et al. (2023)
MG-RAST	S	Api/Web	https://mg-rast.org	Multiple organs Health—disease Diet	Fastq, Fasta, csv, BIOM, JSON	2025	Wide range of studies. Basic ontology annotations. Basic biodiversity metrics are available. Metadata are sometimes missing or not exhaustive.	Meyer et al. (2008)
JGI—IMG/M	S	Api/Web	https://genome.jgi.doe.gov/portal/	Multiple organs Health—disease Diet	Fastq, Fasta, GFF, csv, JSON	2025	Wide range of studies. Basic metadata annotations. Registration is needed for API access.	Chen et al. (2023) and Mukherjee et al. (2023)
gutMDisorder	S	Web	https://bio-computing.hrbmu.edu.cn/gutMDisorder/	Gut Health—disease Diet	csv	2022	Important to obtain the combined effect of disease and nutrients on gut microbiome composition. API is not available.	Qi et al. (2022)
GMrepo	S	Api/Web	https://gmrepo.humangut.info/home	Gut Health—disease	Fastq, JSON, csv	2020	Important to reveal the pangenome and marker taxa of the gut microbiome associated with phenotypes/diseases of other organs. API is not available.	Dai et al. (2022)
Human Gut Microbiome Atlas	S	Web	https://www.microbiomeatlas.org/	Gut/Oral Health—Disease	csv	2022	Possibility of data elaboration integrating microbiome taxonomy composition, disease, and geography. Results are visible only on the GUI, but not available for download.	NA
NIBN JMD	S	Web	https://jmd.nibn.go.jp/	Gut Health—diet	GUI visualization	2025	Fully accessible only through an email request. The results are visible only on the GUI but are not available for download.	Chen Y.-A. et al. (2025)
GIMICA	S	Web	https://gimica.idrblab.net/ttd/	Multiple organs Health—disease	csv	2020	Valuable resource for human genetic and immune factors regulating the microbiome. Data integration is not foreseen.	Tang et al. (2021)
DISBIOME	S	Api/Web	https://disbiome.ugent.be/home	Multiple organs Health—disease	JSON	2018	All embedded data are structured in JSON format. Easy to use in routine bioinformatics pipelines. The data are outdated.	Janssens et al. (2018)
gcMeta	S	Web	https://gcmeta.wdcm.org/	Multiple organs (gut, skin, vaginal)	csv	2025	Contains MAGs from different environments. Results within the DB can be compared and integrated. Data retrieval is challenging from the GUI as the API is absent. Not all data can be downloaded (e.g., functional features).	Shi et al. (2019)
Microbiome database (MDB)	S	Web	https://db.cngb.org/microbiome/	Multiple organs Health—disease	Fasta, csv	2025	It provides genes, MAGs, taxonomic, and functional profiles associated with different biomes. API is not available.	NA
Qiita	S	Api/Web/ Qiime2	https://qiita.ucsd.edu/	Multiple organs Health—disease Diet	BIOM, csv	2025	Accessible through pre-defined tools (Qiime2, redbiom). It needs advanced technical skills. Data integration and comparison are possible.	Gonzalez et al. (2018)
curatedMetagenomicData	S	R-Package	https://waldronlab.io/curatedMetagenomicData/	Multiple organs Health—disease	R/python objects	2021	Accessible through pre-defined tools (R, Python, Docker). It needs advanced technical skills. Data integration and comparison are possible.	Pasolli et al. (2017)
Phenotype database	S	API/Web	https://dashin.eu/interventionstudies/	Multiple organs Health—disease Diet	JSON, csv	2025	Important architecture for food ontology annotation and data integration. It would need automation for data/metadata upload and annotation.	van Ommen et al. (2010)

MAG, metagenome-associated genome; ftp, file transfer protocol; GUI, graphical user interface; JSON, JavaScript Object Notation; csv, comma-separated values; BIOM, Biological Observation Matrix format; NA, not available; P, primary; S, specialized.

Although all of the above resources have a significant level of data curation, integration, and annotation, studies with structured metadata related to food, diet, and their nutritional characteristics associated with health are only present in a few databases (Phenotype Database, NIBN JMD, and gutMDisorder) as such information are usually poorly highlighted and represented as free text format in experimental design description or in the accompanying scientific publications whenever present. Lacking some essential structured information denotes a clear limit for data interoperability and accessibility purposes (see Table 1 “notes” column). Accordingly, essential metadata should be enriched and included at the data submission step through pre-designed templates. Such templates should consult specific ontologies describing the samples comprehensively and their belonging to the study. Enriched metadata studies, when incorporated into frameworks with FAIR characteristics, would offer a concrete and important possibility for data reuse, studies alignment, and advanced scientific discoveries.

## 4 Main databases characteristics

Content characteristics and volume, data and metadata formats/standards, bioinformatics analysis availability, and FAIR-compliance were the criteria to select and describe three databases in the context of microbiome related to food and health. Accordingly, in the following, the main features of Mgnify, MG-RAST, and JGI databases are illustrated.

All three databases provide enhanced accessibility through either a Graphical User Interface (GUI) or through a dedicated Application Programming Interface (API), allowing the incorporation of the resource into standardized data analysis pipelines.

Data retrieval allows access to different metadata categories that contextualize sequencing experiments and describe various types of information about the sample’s origin using ontological terms, sequencing protocols, analytical parameters, and the underlying bioinformatic workflows used for data elaboration. The outputs are provided primarily in JavaScript Object Notation (JSON) format, easily extensible and transformable into widely used standard formats (e.g., csv—comma-separated values).

MGnify *(formerly EBI-Metagenomics)* is a freely accessible platform for assembling, analyzing, and storing metagenomic data from a wide range of environmental and host-associated samples. It supports the analysis of amplicon sequencing (metabarcoding), metagenomics, and metatranscriptomics through standardized, versioned bioinformatic pipelines optimized for different sample types. Multiple pipeline versions are available in a public repository on GitHub,^4^ allowing data reprocessing according to the latest update and straightforward alignment and comparison across different studies’ results. MGnify integrates with external repositories, such as the European Nucleotide Archive (ENA), to enhance data interoperability and reusability and studies metadata tracking, including study unique accession, experiment description (free text), the samples’ biome of origin and links to geographical coordinates, related scientific papers, analyses results, and download pages (example of data retrieval output is available in Supplementary Table 1).

Similarly, MG-RAST is an open-source platform for metagenomics data functional and taxonomic profiling, with additional statistical metrics (alpha diversity index). It contains information regarding *MIxS data* standards compliance (Yilmaz et al., 2011) and metadata classification based on Environmental Ontology (ENVO) (Buttigieg et al., 2013), such as biome_id and material_id, offering the possibility of studies/datasets reuse and potential cross-alignment with other databases (for more details and a complete list of metadata, see Supplementary Table 2). It also uses the information in the Metagenome Annotation Information Resource database (M5nr, Wilke et al., 2012) to harmonize functional annotations and biochemical pathways from multiple sources (i.e., GenBank, UniProt, KEGG, and SEED).

*JGI Data Portal* is a centralized repository for storing raw (also connected to SRA) and annotated genomic and metagenomic data from microbial communities, plasmids, viruses, and fungi (typical retrieval output details in Supplementary Table 3). Through the Genomes Online Database (GOLD) (Mukherjee et al., 2023), JGI organizes structured MIxS-compliant metadata, as well as MIGS/MIMS and MIxE standards (Field et al., 2008; Yilmaz et al., 2011). A key component is the *Integrated Microbial Genomes & Microbiomes (IMG/M)* system, which enables comparative genomic analysis and functional annotation of microbial communities through the JGI metagenome workflow and the DOE-JGI Metagenome annotator pipeline (Huntemann et al., 2016; Chen et al., 2023).

## 5 Discussion

The integration and alignment of metagenomic data related to diet, nutrition, and health present a critical challenge in human microbiomics research. While specific projects and their dedicated data resources (i.e., HMP, GMrepo, GIMICA, DISBIOME, and gcMeta) are focused on a defined scientific area, larger databases (i.e., Mgnify, MG-RAST, and JGI) aggregate large volumes of data across multiple scientific disciplines, including diet and/or health targeting the microbiome of multiple organs (Table 1). These encompass either observation (e.g., microbiome profiling of obese or diabetic subjects, healthy individuals, disease-related functionally/differentially expressed biome, and geographically tagged microbiome) or intervention studies (including time-series experiments on specific food intake or medicinal treatment). Structure and comprehensiveness of metadata related to such studies are crucial to understand the experiment itself and to provide insights into the scientific area of interest, helping the improvement and evolution of experimental designs with similar objectives and avoiding replicating research.

Accordingly, the adherence to specific data and metadata standards [e.g., MIxS (Field et al., 2008; Yilmaz et al., 2011)] and the unification of bioinformatics pipelines, as suggested by FAIR data principles (Wilkinson et al., 2016), (see text footnote 1) would offer an important asset toward data discovery, harmonization across platforms, interoperability, accurate reuse, and effective metadata management (Vitali et al., 2018; Balech et al., 2022). In this context, the use of ontologies (disease and/or food) as a unified and standardized language is a vital step toward the implementation of *FAIR* principles, ensuring a consistent representation of complex data and facilitating the application of novel technologies (i.e., AI-driven data mining and classification) to connect various systems and platforms. For instance, linking Mgnify, MG-RAST, and JGI studies through unique identifiers or ontology terms, such as biosampleID, bioprojectID, or ENVO classes, would offer a concrete possibility to merge the corresponding data to obtain broader scientific research hypotheses.

The Ontology Lookup Service (OLS, Côté et al., 2010),^5^ of EBI or the ontologies available through BioPortal (Noy et al., 2009),^6^ provide a framework where it is possible to use multiple ontologies that could facilitate the classification of metagenomic studies data and associated metadata based on biological, ecological, functional, nutritional, and health parameters. Leveraging these ontologies and recognizing their importance to maximize research outputs can lead to their further development, which will promote consistent and innovative changes in shaping diet and microbiome research by proposing new experimental designs.

Another key aspect in this context is the integration of sequencing data with dietary patterns and disease conditions. Traditional microbiome studies often emphasize broad taxonomic classifications, but recent advances in functional metagenomics highlight the need to link microbiome data with specific biochemical pathways and metabolic functions. As seen in Table 1, databases such as GIMICA (Tang et al., 2021) or DISBIOME (Janssens et al., 2018) provide an enhanced view of disease or genetic-immunological factors that can shape a healthy microbiome in different organs. However, such data would be additionally comprehensive while linking those aspects to lifestyle and dietary habits. By associating microbiome datasets with dietary interventions based on nutritional intake and health biomarkers, we gain deeper insights into how microbial communities influence metabolic pathways, immune responses, and disease progression. A valuable example of such an approach is the DASH-IN initiative (Data Sharing in Nutrition)^7^ (van Ommen et al., 2010), associated with Phenotype Database, that promotes data integration and sharing in nutritional research across platforms, while offering the possibility to annotate studies metadata using ontological terms to capture the complexity of experimental designs and fulfill the requirements of FAIR data principles. Similarly, the availability of structured and analyzed cohort studies of gut microbiome data with extensive metadata (diet, nutrient intake, physical activity) in databases such as gutMDisorder (Qi et al., 2022) and NIBN JMD (Chen Y.-A. et al., 2025) represents a promising starting point toward building ready-to-use data integration and harmonization schemas spanning across other organs or disease-related factors. Therefore, comprehensive ontologies that annotate diets at different levels (dietary pattern, food group, food components) are important to understand the true relationships between diet and health mediated by the gut microbiome.

**Figure 1:**
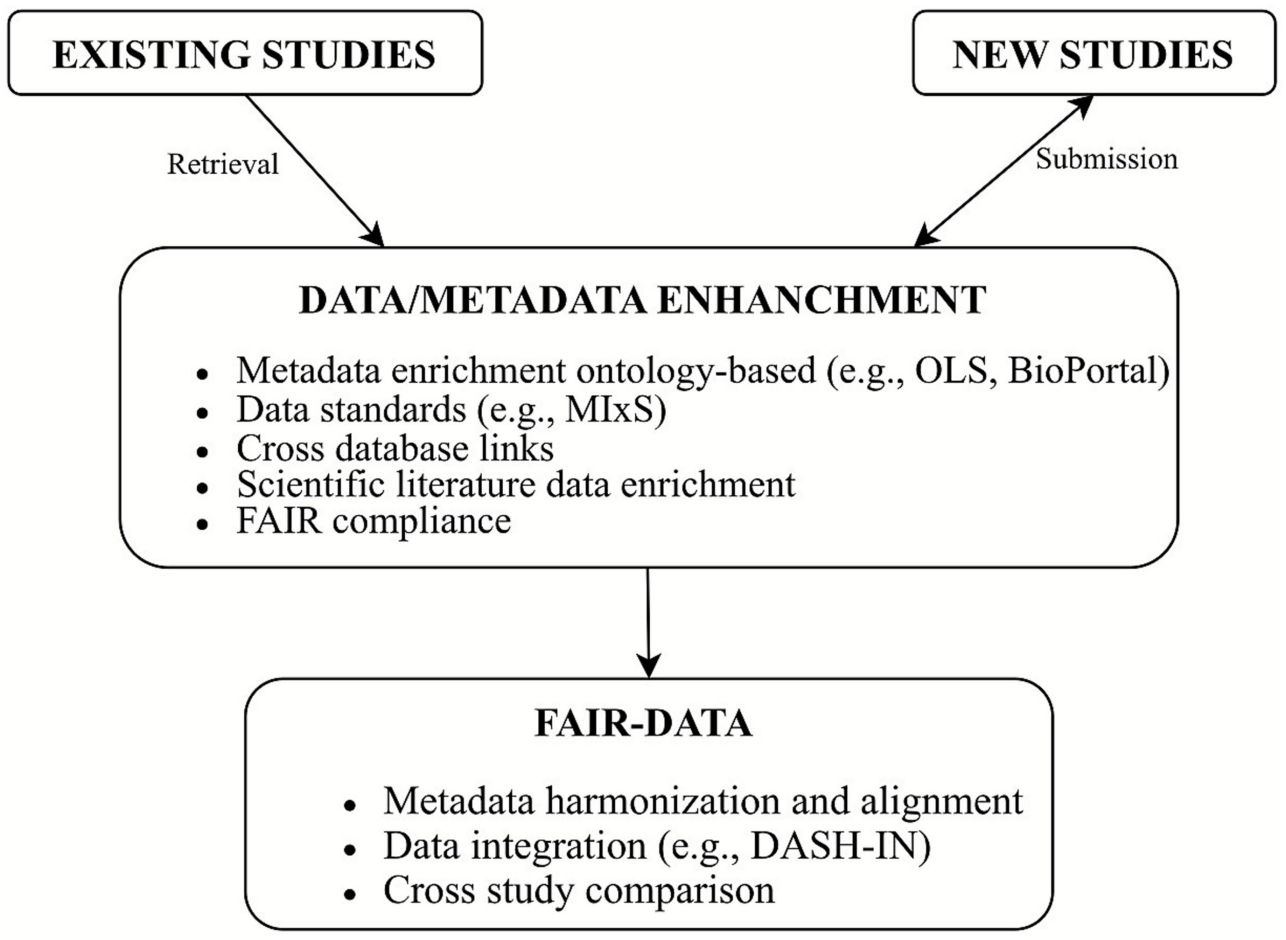
Conceptual workflow on microbiome data and metadata FAIRfication and enrichment. The proposed implementation steps are illustrated for both new data submissions and those already present in the source databases.

As stated above, the perspectives to obtain rich microbiome data and metadata resources are summarized in Figure 1, which describes shortly the main workflow to be implemented as an integral part of the new data submission or enrichment system of studies already present in the source databases. Similar concepts, including technical information technology steps, were also proposed recently, illustrating potential actions to be fulfilled at the submission level (Hug et al., 2025; Speir et al., 2025). Although similar, the approach proposed in this review (Figure 1) highlights the potential strength of open science concept assessment and advanced technology annotations to exploit both existing and new microbiome studies.

An urgent need, however, lies in the implementation of a combined methodology exploiting state-of-the-art technologies to mine information embedded not only in primary or specialized databases but also in scientific literature. This has become increasingly possible with the use of data science and natural language processing for semantic annotation and searching of relevant metadata information. Such approaches, if adopted, introduce scientific research into the new era of technology enhanced by information management and strengthen decision-making dealing with microbiome-targeted therapies for health and nutrition.

## Footnotes

1
https://faircookbook.elixir-europe.org/content/home.html

2
https://db.cngb.org/microbiome/

3
https://www.microbiomeatlas.org/

4
https://github.com/EBI-Metagenomics

5
https://www.ebi.ac.uk/ols4/

6
https://bioportal.bioontology.org/

7
https://dashin.eu/interventionstudies/
